# Impact of Polymer Backbone Fluorination on the Charge Generation/Recombination Patterns and Vertical Phase Segregation in Bulk Heterojunction Organic Solar Cells

**DOI:** 10.3389/fchem.2020.00144

**Published:** 2020-03-05

**Authors:** Yanqiu Shao, Yuying Chang, Suju Zhang, Mingyue Bi, Shengjian Liu, Daliang Zhang, Shirong Lu, Zhipeng Kan

**Affiliations:** ^1^School of Chemistry and Chemical Engineering, Mudanjiang Normal University, Mudanjiang, China; ^2^Heilongjiang Province Key Laboratory of New Carbon-Base Functional and Superhard Material, Mudanjiang, China; ^3^Guangzhou Key Laboratory of Materials for Energy Conversion and Storage, Guangdong Provincial Engineering Technology Research Center for Materials for Energy Conversion and Storage, School of Chemistry, South China Normal University (SCNU), Guangzhou, China; ^4^Institute of Advanced Interdisciplinary Studies, Chongqing University, Chongqing, China; ^5^Organic Semiconductor Research Center, Chongqing Institute of Green and Intelligent Technology, Chinese Academy of Sciences, Chongqing, China

**Keywords:** bulk heterojunction, polymer backbone fluorination, charge generation and recombination, vertical phase segregation, organic solar cells

## Abstract

Incorporating fluorine (–F) substituents along the main-chains of polymer donors and acceptors is an effective strategy toward efficient bulk-heterojunction (BHJ) solar cells. Specifically, F-substituted polymers often exhibit planar conformations, leading to favorable packing, and electronic coupling. However, the effects of fluorine substituents on the charge generation and recombination characteristics that determine the overall efficiency of BHJ active layers remain critically important issues to examine. In this report, two PBDT[2X]T polymer analogs –poly[4,8-bis((2-ethylhexyl)oxy)benzo[1,2-*b*:4, 5-*b*′]dithiophene-thiophene] [PBDT[2H]T] and its F-substituted counterpart poly[4,8-bis((2-ethylhexyl)oxy)benzo[1,2-*b*:4,5-*b*′]dithiophene-3,4-difluoro-thiophene] [PBDT[2F]T]—are studied to systematically examine how –F substituents impact the blend morphology, charge generation, carrier recombination and extraction in BHJ solar cells. Considering the large efficiency differences between PBDT[2H]T- and PBDT[2F]T-based BHJ devices, significant emphasis is given to characterizing the out-of-plane morphology of the blend films as vertical phase-separation characteristics are known to have dramatic effects on charge transport and carrier extraction in polymer-fullerene BHJ solar cells. Herein, we use electron energy loss spectroscopy (EELS) in tandem with charge transport characterization to examine PBDT[2X]T-fullerene blend films. Our analyses show that PBDT[2H]T and PBDT[2F]T possess very different charge generation, recombination and extraction characteristics, resulting from distinct aggregation, and phase-distribution within the BHJ blend films.

## Introduction

The substitution of π-conjugated polymer chains with fluorine (–F) substituents is an effective strategy in the design of polymer donors and acceptors for efficient bulk-heterojunction (BHJ) solar cells (Li et al., 2019). Of all considered benefits, recent studies have shown that F-substituted polymers are prone to adopt planar conformations, and favorable packing and electronic coupling patterns (Do et al., 2016; Tang et al., 2020) broader consensus emphasizes their propensity for higher backbone rigidity and a more pronounced tendency to aggregate on going from solutions to thin-film (Li et al., 2014, 2020; Fei et al., 2015) compared to their counterparts without –F substituents –material properties that can be taken advantage of in BHJ solar cell optimization processes (Liu et al., 2014).

In spite of the significant experimental work pursued to describe the correlations between –F substitutions in polymer main-chains and polymer performance in actual BHJ solar cells, direct connections between material structure and device efficiency are often difficult to make. Considering F-substituted polymers, some important questions remain, in particular with regard to how molecular scale effects directed by –F substituents can impact mesoscale morphologies, charge generation, and recombination patterns in BHJ thin films (Eisner et al., 2019). Since experimental methods are lacking to directly probe how functional group substitutions affect polymer main-chain packing and aggregation, computational methodologies must be employed to provide this level of description (Do et al., 2017). Quantum mechanical calculations can describe intrinsic *inter-*monomer torsion profiles, which govern the main-chain dihedral distribution in the bulk; whereas molecular dynamics (MD) simulations can provide information on main-chain packing arrangements in the bulk, and the propensity to form ordered π-π aggregates (Li et al., 2020). To further molecular-scale insights and qualitatively describe the types of aggregates that prevail in the bulk, solid-state nuclear magnetic resonance (SS-NMR) spectroscopy can be used to complement computational methodologies, providing an experimental analysis of the conformational landscape defined by the polymer main-chains in the bulk (Do et al., 2016). Based on this methodology, both theoretical and experimental studies on the influence of –F substitutions on *intra*- and *inter-*molecular interactions have shown that the higher polymer backbone planarity and rigidity are at the origin of local packing effects (to which relate binding energies and electronic coupling between neighboring chains), and that these result in distinct aggregation and charge transport patterns (Do et al., 2016). As an example, in poly(3-alkyl-4-fluoro)thiophenes (F-P3AT) (Fei et al., 2015) –F substitutions have been shown to raise the melting temperature and crystallization enthalpy of the P3AT analogs –variations in intrinsic material properties that are consistent with backbone planarization effects, inducing main-chain rigidity, and higher propensity for aggregation in the solid state. In turn, employing F-P3AT, field-effect transistor mobilities increased by a factor of 5 compared to P3AT-based transistors (Fei et al., 2015).

In polymer-based BHJ solar cells, –F substitutions of monomer units including: thiophenes (Jo et al., 2014; Wolf et al., 2015), carbazoles (Kim et al., 2014), thienothiophenes (Chen et al., 2009; Carsten et al., 2011), benzothiadiazole (Stuart et al., 2013; Yang et al., 2013; Kim et al., 2014), benzotriazoles (Price et al., 2011; Chen et al., 2019), benzodithiophenes (Chen et al., 2009; Jo et al., 2014; Wolf et al., 2015), indacenodithiophenes (Schroeder et al., 2012), and anthradithiophenes (Gundlach et al., 2008) have resulted in improved device efficiencies. Due to the strong inductive electron-withdrawing nature of –F substituents (element of highest electronegativity), F-substituted polymer donors possess lower-lying highest occupied molecular orbital (HOMO) energy levels than their counterparts without –F substituents –leading to higher open-circuit voltages (*V*_*OC*_) in BHJ solar cells (Chen et al., 2009, 2019). Setting aside their important effect on the electronic properties of both polymer donors (Chen et al., 2009; Price et al., 2011; Schroeder et al., 2012; Stuart et al., 2013; Yang et al., 2013; Jo et al., 2014; Kim et al., 2014; Li et al., 2014; Wolf et al., 2015; Do et al., 2016; Kawashima et al., 2016) and acceptors (Liu et al., 2016, 2017), –F substitutions may also affect the preferential orientation of polymer aggregates relative to device substrates, impacting device performance in some instances (Stuart et al., 2013), although here we note that those effects are not well-understood to date, and there were reports with opposite conclusions (Kawashima et al., 2016). Most systematic studies of the effect of F-substituents in polymers (comparing F-substituted vs. unsubstituted, analogous model systems) have converged to the idea that device performance can vary dramatically when either donor or acceptor contain F-substituents in their main-chain (Jo et al., 2014; Wolf et al., 2015; Kawashima et al., 2016). Along those lines, several reports have shown that concurrent synthetic and device optimizations of fluorinated polymers can yield significant improvements in BHJ thin films as carrier mobilities increase, and that those improvements result in direct BHJ solar cell efficiency increments (Jo et al., 2014; Wolf et al., 2015). Using pump probe ultrafast spectroscopy, Gorenflot et al. (2018) reported the exciton dissociation, charge separation, and extraction in BHJ solar cells with fluorine substituents, and it was found that in the fluorinated polymer BHJ solar cells, the charge generation is about 40% higher than that of the non-fluorinated polymer BHJ solar cells, resulting from improved exciton diffusion to the heterojunction, in conjunction with more efficient charge separation and reduced geminate recombination losses (Gorenflot et al., 2018). However, the effects of the presence of F-substituted polymers on the charge generation and recombination characteristics in BHJ solar cells, and how those effects translate into practical device efficiency variations, remain critically important issues to examine. In addition, significant emphasis is given to characterize the “out-of-plane” morphology of the blend films, because vertical phase-separation characteristics are known to have dramatic effects on charge transport and carrier extraction in polymer-fullerene BHJ solar cells.

**Chart 1:**
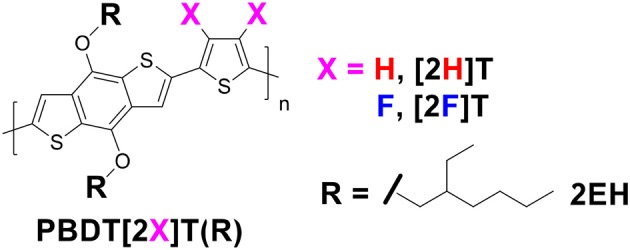
Chemical structures of the PBDT[2X]T polymers (with X = H or F).

In this report, we examine the charge generation and recombination in two analogous model systems in BHJ solar cells: poly[4,8-bis((2-ethylhexyl)oxy)benzo[1,2-*b*:4,5-*b'*]dithiophene-thiophene] [PBDT[2H]T] and its F-substituted counterpart poly[4,8-bis((2-ethylhexyl)oxy)benzo[1,2-*b*:4,5-*b*′]dithiophene-3,4-difluoro-thiophene [PBDT[2F]T]. (Chart 1) The PBDT[2X]T polymers dispersion index and molecular weight are shown in Table S3. Specifically, we systematically characterize carrier transport, recombination, and extraction across the BHJ devices and turn to morphological studies to establish a fuller understanding of how aggregation and vertical phase distributions impact the carrier dynamics, and in turn, BHJ solar cell efficiency.

## Results and Discussion

**Figure 1:**
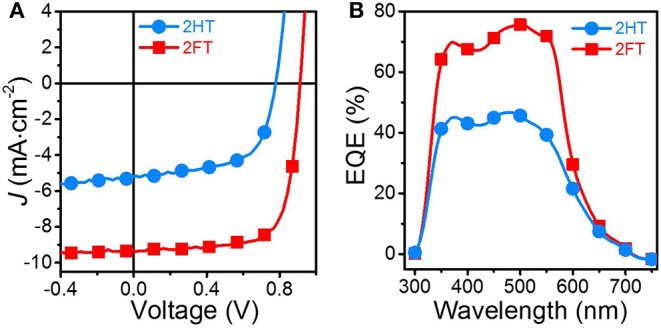
**(A)** *J-V* curves and **(B)** EQE spectra for optimized 2HT- (blue) and 2FT-based (red) BHJ solar cells with PC_71_BM as the acceptor; AM1.5G solar illumination (100 mW/cm^2^).

Optimized BHJ solar cells with direct device architecture were fabricated and tested under AM1.5G solar illumination (100 mW/cm^2^). The PBDT[2X]T:PC_71_BM blend solutions (ratio: 1:1.5, wt/wt) were cast from a hot chlorobenzene (CB; *ca*. 90°C) solution with 5 vol% 1-chloronaphthalene (CN) (cf. details in the SI; film thicknesses in the range 80–90 nm). The energy level diagram of PBDT[2H]T, PBDT[2F]T and PC_71_BM in Figure S2. Figure S3 accounts for the relative thin-film absorbance and solution absorption coefficients of the PBDT[2H]T and PBDT[2F]T. As shown in Figure 1A and Table 1, the optimized PBDT[2H]T:PC_71_BM (2HT-based) and PBDT[2F]T:PC_71_BM (2FT-based) BHJ solar cells achieved very distinct efficiencies and device statistics including standard deviations are provided in the Supplementary Information (Tables S1, S2; Figure S1). While the optimized 2HT-based devices achieved *V*_*OC*_ of *ca*. 0.77 V, modest *J*_*SC*_of *ca*. 5.2 mA/cm^2^, and fill-factors (*FF*s) of 60.1%, the 2FT-based counterpart yields a substantially higher *J*_*SC*_ of *ca*. 9.3 mA/cm^2^, and a concurrently improved *FF* (73.3%) and *V*_*OC*_ (*ca*. 0.89 V). Overall, 2FT-based devices achieve a power conversion efficiency (PCE) improvement of more than two-fold, reaching up to 6.2% (*avg*. 6.1%) under the same film-casting conditions.

**Table 1:** PV performance of the 2HT- and 2FT-based BHJ solar cells^a^^,^ ^b^.

	***V*_**OC**_ (V)**	***J*_**SC**_ (mA/cm^**2**^)**	***FF* (%)**	**Avg. PCE (%)**	**Max. PCE (%)**
2HT	0.77	5.2	60.1	2.4	2.7
2FT	0.89	9.3	73.3	6.1	6.2

a Average values across >10 devices across 3 substrates.

b *Device statistics in the Supplementary Information, Table S1*.

The large differences in *J*_SC_ values between 2HT- and 2FT-based BHJ solar cells (Table 1) are shown in the *J-V* curves provided in Figure 1A, and are consistent with the integrated current from the external quantum efficiency (EQE) spectra shown in Figure 1B (± 6%). In Figure 1B, 2FT-based devices show the most prominent spectral response in the range 350–600 nm, with EQE values reaching *ca*. 75% at 500 nm (EQE >60% in the range 350–580 nm). In comparison, EQE values in 2HT-based devices remain below 50% across the visible spectrum –observations implying that the substitution pattern in PBDT[2X]T correlates with significant variations in BHJ solar cell performance.

The competition between charge recombination and extraction governs the *FF* in BHJ solar cells and in turn device PCE. Meanwhile, both the loss of photogenerated carriers by recombination and charge extraction processes are limited by the carrier mobilities of the blend film for holes and electrons. To estimate the electron and hole mobilities in optimized PBDT[2X]T:PC_71_BM blend films, we measured the dark current density-voltage characteristics of single-carrier devices (see experimental details in the SI) and then fitted the data using the space-charge-limited current (SCLC) (Giulianini et al., 2010) model described by the Mott-Gurney law (with a small field dependent term), as in following equation (Mihailetchi et al., 2005).

(1) $$\begin{array}{l} J\left(V\right)=\frac{9}{8}\varepsilon_{0}\varepsilon_{r}\mu_{0}\exp\left(0.89\beta \sqrt{\frac{V-V_{bi}}{L}}\right)\frac{{\left(V-V_{bi}\right)}^{2}}{L^{3}} \end{array}$$

where ε_0_ and ε_*r*_ are the dielectric permittivity of a vacuum and the active layer, *L* is the thickness of the active layer, *V* is the applied voltage, *V*_*bi*_ is the built-in voltage, μ_0_ is the zero-field mobility, and β is the field-activation factor.

**Figure 2:**
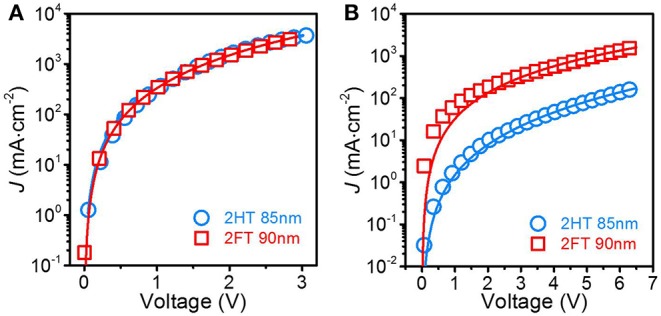
Dark current density-voltage characteristics for **(A)** electron-only (ITO/Al/active layer/Al) and **(B)** hole-only (ITO/MoO_3_/active layer/MoO_3_/Ag) diodes with optimized PBDT[2X]T:PC_71_BM active layers.

Figure 2 shows the dark *J*-*V* characteristics of the electron-only diode in the configuration ITO/Al/2XT:PC_71_BM/Al (Figure 2A) and that for the hole-only diode with the architecture ITO/MoO_3_/2XT:PC_71_BM/MoO_3_/Ag (Figure 2B) (cf. additional details in the Supplementary Information, Figure S4). The fitting to the experimental results (solid lines in Figure 2; the parameters used in the fitting and films with other thickness are detailed in the Supplementary Information, Figure S4, Tables S4 and S5) indicate that the electron mobility in the BHJ blend films with the 2HT and 2FT polymers is comparable: *ca*. 6.2 × 10^−4^ cm^2^ V^−1^s^−1^ and 6.8 × 10^−4^ cm^2^ V^−1^s^−1^, respectively. In contrast, the hole mobility in the 2HT- and 2FT-based blend films are an order of magnitude different: *ca*. 4.4 × 10^−6^ vs. 3.5 × 10^−5^ cm^2^ V^−1^s^−1^, respectively. As a result, the carrier mobilities are significantly more balanced in 2FT-based devices –which represents an important parameter in explaining the performance differences observed between 2HT- and 2FT-based BHJ solar cells.

The charge collection probability, *P*_*c*_(*I, V*), is defined as the ratio between *J*_*SC*_ and saturated photocurrent density *J*_*ph*.*sat*._ at reversed biases when the current density becomes independent of the applied voltage (cf. details in the Supplementary Information, Figures S5, S6) (Cowan et al., 2010; Mori et al., 2014).

**Figure 3:**
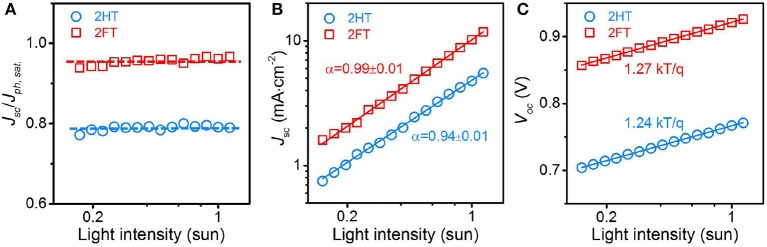
**(A)** Charge collection probability *P*_*c*_ as a function of incident light intensity in linear-log scale, **(B)** *J*_*SC*_ as a function of incident light intensity in log-log scale, and **(C)** *V*_*OC*_ as a function of incident light intensity for the 2HT- and 2FT-based BHJ solar cells in linear-log scale. The solid lines in **(B,C)** correspond to fits to the data based on the equations $J_{sc}\propto I^{\alpha }$ and $V_{oc}\propto n\frac{kT}{q}\ln\left(I\right)$, respectively.

Figure 3A shows the *P*_*c*_(*I*, −2*V*) for the 2HT- and 2FT-based BHJ solar cells as a function of incident light intensity *I*. In both cases, the *P*_*c*_ values were almost independent of incident light intensity over the range 0.17–1.20 suns, yielding *ca*. 80% for 2HT-based devices and 95% for their 2FT counterparts. When the charge carrier density increases with increasing light intensity, the magnitude of the bimolecular recombination rate should also increase and the *P*_*c*_ should decrease at higher values of *I* if the device suffers non-negligible bimolecular recombination at short-circuit. Therefore, the independence of *P*_*c*_ on *I* suggests that neither 2HT- nor 2FT-based devices suffer from bimolecular recombination under the short-circuit conditions.

To further examine whether carrier recombination was limiting device efficiency to a different extent in 2HT- and 2FT-based devices, we turned to a characterization of *J*_*SC*_ and *V*_*OC*_ as a function of incident light intensity. Figure 3B provides the dependence of *J*_*SC*_ as a function of incident light intensity plotted in a log-log scale and fitted to a power law (solid lines). As reported in earlier work, a power law dependence of *J*_*SC*_on incident light intensity *I* in BHJ solar cells is described by $J_{sc}\propto I^{\alpha }$ (Cowan et al., 2010; Koster et al., 2011; Kyaw et al., 2013). An exponential factor of α = 1 (or near unity) is indicative of efficient carrier extraction prior to recombination at short-circuit. For 2HT-based devices, the power law fit to the *J*_*SC*_ vs. *I* data yields an α value of 0.94 ± 0.01, while for 2FT-based devices, the *J*_*SC*_ vs. *I* data fit yields an α value of 0.99 ± 0.01, indicating that carrier extraction proceeds with more recombination losses in 2HT-based BHJ solar cells. Therefore, through the analysis of *P*_*c*_ and the *J*_*SC*_ dependence on incident light intensity, 2HT-based devices suffer from two major losses channels thus far: (i) a relatively poor charge generation pattern reflected in the low *J*_*SC*_ (6 mA/cm^−2^) and (ii) non-negligible recombination losses prior to extraction.

In parallel, Figure 3C depicts the variation of *V*_*OC*_ vs. *I* in a natural log-linear scale and fitted to $V_{oc}\propto n\frac{kT}{q}\ln\left(I\right)$ (Cowan et al., 2010; Kyaw et al., 2013), where *k*, *T*, and *q* are the Boltzmann constant, temperature in Kelvin, and the elementary charge, respectively. The parameter *n* (usually in the range of 1–2) accounts for the presence of carrier traps across the active layers or interfaces with the electrodes, and any deviations from *n* = 1 (trap-free condition) reflects the existence of trap-assisted recombination. As shown in Figure 3C, *n* = 1.24 and 1.27 were inferred for 2HT- and 2FT-based BHJ solar cells, respectively –implying that both 2HT- and 2FT-based devices suffer from trap-assisted recombination at open-circuit. To further our understanding of the charge recombination and extraction patterns in 2HT- and 2FT-based BHJ solar cells, we turned to transient photovoltage and photocurrent analyses.

Transient photovoltage (TPV) measurements and their analysis provide information regarding the non-geminate recombination of charges within devices. TPV measurements record the voltage decay transient of a device held at open-circuit under continuous illumination after being subject to a short perturbative light pulse; the photovoltage decay can be used as a direct measure of charge recombination kinetics in BHJ solar cells at open-circuit. One or two time constants, representative of carrier lifetime, can be obtained by fitting the decay kinetics with mono- or bi-exponential equations, suggesting that one or two recombination routes can co-exist, that can generally be assigned to either bimolecular or/and trap-assisted recombination (Li et al., 2011; Liang et al., 2018). TPV measurements were performed on the 2HT- and 2FT-based BHJ solar cells to characterize the non-geminate recombination profile in the two systems.

**Figure 4:**
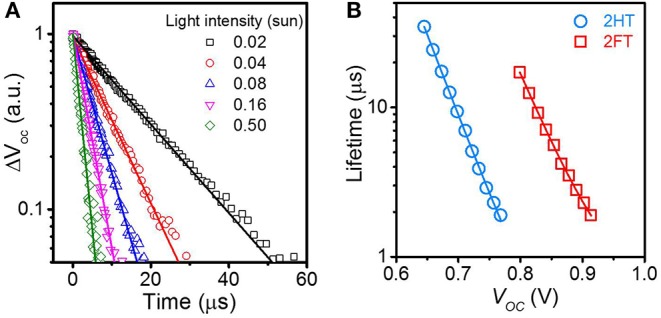
**(A)** Normalized transient photovoltage perturbation from 2FT-based BHJ solar cells under various light intensities at open-circuit. **(B)** Carriers lifetime as a function of *V*_*OC*_ from the best performed device. The solid lines in both figure subsets are mono-exponential fits (cf. fitting details in the SI).

In both 2HT- and 2FT- based BHJ solar cells, mono-exponential fits to the experimental kinetics data were found to be appropriate fitting equations. Figure 4A shows the normalized TPV responses for a 2FT-based device subjected to several incident light intensities (Data fitting details reported in the Supplementary Information, see Figure S7). The single carrier lifetime figure derived from the TPV fitting of 2HT- and 2FT-based devices correlate with a single carrier recombination loss channel, here trap-assisted recombination as determined from the variation of *V*_*OC*_ with illumination intensity discussed in the previous section. Figure 4B plots the carrier lifetime as a function of *V*_*OC*_ for the 2HT- and 2FT-based BHJ solar cells. As expected, the carrier lifetime decreases with increasing *V*_*OC*_, and the carrier lifetime at 1 sun is estimated as 2.3 and 2.0 μs (comparable) for 2HT- and 2FT-based devices, respectively.

Carrier transport and extraction across BHJ active layers can be examined via transient photocurrent (TPC) measurements (Hwang et al., 2009; McNeill et al., 2009; Li and McNeill, 2011; Li et al., 2011, 2013; Gao et al., 2014; Tremolet de Villers et al., 2014; Liang et al., 2019); analyses that are particularly relevant to the study of carrier traps and trap-assisted recombination in BHJ solar cells. In these analyses, the occurrence and extent of carrier traps across the active layer and/or at the interfaces between the BHJ blend film and the electrodes are reflected in the dependence of device current response and shape on light intensity. Instances of carrier traps concentrated at BHJ active layer/electrode interfaces and impinging on charge extraction have been discussed in recent studies (Hwang et al., 2009; McNeill et al., 2009; Li and McNeill, 2011; Tremolet de Villers et al., 2014). For example, in direct BHJ device configurations, fullerenes accumulating at the anode induce transient current peaks, greatly surpassing the steady-state current during rise events. Using concurrent experimental results and theoretical models, it has been shown that fullerenes act as hole-blocking layers, hindering hole extraction at the anode and, in turn, lowering BHJ solar cell efficiency (Tremolet de Villers et al., 2014). Here, we examine the turn-on and turn-off dynamics of the 2HT- and 2FT-based devices using long light pulse excitations (200 μs; cf. details in the SI), allowing the current density to reach steady-state conditions.

**Figure 5:**
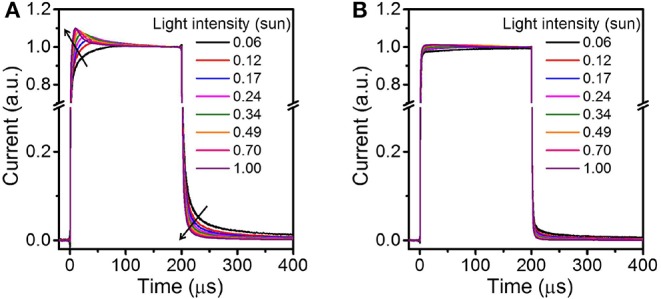
Transient photocurrent (normalized) in response to a 200 μs white light (LED) pulse for **(A)** 2HT- and **(B)** 2FT-based BHJ solar cells. The legend in **(A)** provides the various light intensities (in equivalent suns). The black arrows emphasize the dependence of the photocurrent as a function of light intensity (after pulse excitation) and light intensities (cf. details in the SI).

**Figure 6:**
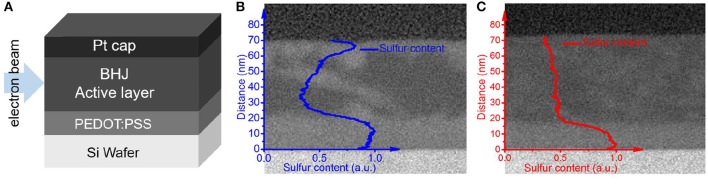
**(A)** Schematic of the samples prepared for the cross-sectional TEM and EELS analyses. TEM cross-section images of the **(B)** 2HT- and **(C)** 2FT-based BHJ active layers cast on PEDOT:PSS and superimposed core-level sulfur analyses for the phase ratio mapping of polymer-rich (brighter regions) and fullerene-rich (darker regions) phases in the BHJ cross-section. The sulfur-content lines vs. distance from the substrate represented in subsets **(B,C)** capture the sulfur distribution averaged across the whole film cross section, and the distances are reported from the surface of the Si wafer (brightest layer) to the Pt capping layer (black).

Figure 5 depicts the normalized transient photocurrent for optimized 2HT- and 2FT-based devices (cf. conditions given in Table 1); supplementary data can be found in the Supplementary Information (Figure S8). Figure 6B shows that the fast dynamics of the 2FT-based devices contrast with that for 2HT-based devices in Figure 6A. The rise/fall times on the order of 2 μs (i.e., the time required to reach 90% of the maximum current from an initial 10%) obtained from 2FT-based active layers (Figure 6B) are practically independent of light intensity. In comparison, the TPC curves for the 2HT-based devices show a significant dependence on light intensity, with a fast-initial transient photocurrent peak at higher light intensities followed by a second, slower photocurrent decay component leveling off at the steady-state current (at short-circuit) within *ca*. 150 μs (Figure 6A). Comparing the turn-off dynamics of the 2HT- and 2FT-based devices: the fast *ca*. 2 μs decay and the absence of pronounced photocurrent tail observed in 2FT-based active layers contrast with the persistent, light-intensity-dependent photocurrent tail observed in 2HT-based active layers, which suggests that carrier collection is delayed by deep traps for as long as 200 μs in the 2HT-based devices. In general, the slower dynamic component (beyond 205 μs, Figures 5A,B) becomes less prominent as light intensity increases, suggesting that the traps are filled at higher light intensities and, as a result, their impact on charge transport becomes less pronounced (McNeill et al., 2009; Li and McNeill, 2011). Based on earlier discussions and prior reports (Heumueller et al., 2014; Tremolet de Villers et al., 2014; Pearson et al., 2016), the transient peak observed in 2HT-based devices may be the result of morphological effects buried within the active layer or occurring at the electrode interfaces. To probe those effects and provide a qualitative, macroscopic insight into the vertical distribution of polymer- and fullerene-rich domains across the optimized BHJ solar cells, we turned to electron energy loss spectroscopy (EELS) analyses performed from cross-sections of the BHJ thin films imaged by transmission electron microscopy (TEM) (cf. experimental details provided in the SI).

Figure 6 provides the EELS profiles collected from the cross-sections of BHJ thin films (cast from optimized conditions, see Table 1) with PBDT[2H]T (Figure 6B) and PBDT[2F]T (Figure 6C). Obtained from core-level sulfur analyses, the superimposed plots provide relative phase ratio mapping for the sulfur-rich—i.e., polymer-rich—and sulfur-deficient—i.e., fullerene-rich—regions in the BHJ cross-section. Here, we note that PEDOT:PSS interlayers were cast between the Si wafer (substrate) and the BHJ active layer to reproduce the morphology of the photoactive layers as obtained in actual BHJ solar cells; the high sulfur contents observed in the first *ca*. 20 nm distance from the Si wafer are thus consistent with the presence of the PEDOT:PSS. Beyond the PEDOT:PSS interface, the sulfur maps show two distinct phase distribution patterns for optimized 2HT- and 2FT-based BHJ active layers. On the one hand, Figure 6B suggests the existence of a gradient of polymer-rich phases across 2HT-based active layers on going from the PEDOT:PSS interface (anode) to the top interface (cathode in an actual BHJ device), with an apparent peak indicative of an accumulation of the polymer donor 2HT near the top interface (cathode). On the other hand, Figure 6C reflects a relatively uniform concentration profile of polymer and fullerene throughout the depth of the BHJ active layer. If supported by a more quantitative analysis of the vertical phase distribution in the BHJ active layers, the polymer-rich phase concentrated near the top interface (cathode) in 2HT-based active layers can hinder electron extraction by forming an electron-blocking layer. Concurrently, the apparently fullerene-rich phase concentrated near the PEDOT:PSS interface (anode) in 2HT-based active layers (Figure 6B) may be detrimental to hole extraction (hole-blocking layer).

## Conclusion

To summarize, we systematically characterized the charge generation and recombination patterns in BHJ solar cells with PBDT[2H]T and its F-substituted counterpart PBDT[2F]T, and used EELS in tandem with charge transport characterization to examine PBDT[2X]T-fullerene blend films. The reduction in microsecond transient photocurrent allowed us to attribute the recombination losses in the 2HT-based BHJ solar cells to the hole extraction barrier/traps. However, the fluorinated polymer donor 2FT- based photoactive layer had a more uniformly distributed polymer/fullerene blend throughout most of the depth of the film with a thin fullerene accumulation layer at the anode, without showing the reduction in photocurrent. Thus, 2FT-based BHJ solar cells showed more efficient charge generation, extraction, and higher hole mobility compared with those of the 2HT-based BHJ solar cells, leading to higher *V*_*OC*_, *J*_*SC*_, FF, and overall device performance. The vertical phase segregation features of the 2HT- and 2FT-based BHJ solar cells were confirmed with EELS (sulfur mapping). The –F substituents impacted the polymer packing, which translates to the difference in aggregations from solution to film, thus the differences in polymer-fullerene phase mixing/composition in BHJ thin films. Our results provide an insight into the fluorination effects on the thin film BHJ compositions, especially the vertical phase, thus the device efficiency, providing a direct evidence of benefits from fluorination of polymers.

## Data Availability Statement

All datasets generated for this study are included in the article/supplementary material.

## Author Contributions

ZK, YS, YC, and SLu proposed the idea of this paper and contributed to analize the experiment results and wring the paper. YC and ZK contributed to the fabrication of the solar cells and characterization. DZ conducted the TEM. SLi contributed to the synthesis of the donor. SZ and MB conducted SCLC.

### Conflict of Interest

The authors declare that the research was conducted in the absence of any commercial or financial relationships that could be construed as a potential conflict of interest.
