# Corrigendum: Impact of Polymer Backbone Fluorination on the Charge Generation/Recombination Patterns and Vertical Phase Segregation in Bulk Heterojunction Organic Solar Cells

**DOI:** 10.3389/fchem.2020.00267

**Published:** 2020-04-08

**Authors:** Yanqiu Shao, Yuying Chang, Suju Zhang, Mingyue Bi, Shengjian Liu, Daliang Zhang, Shirong Lu, Zhipeng Kan

**Affiliations:** ^1^School of Chemistry and Chemical Engineering, Mudanjiang Normal University, Mudanjiang, China; ^2^Heilongjiang Province Key Laboratory of New Carbon-Base Functional and Superhard Material, Mudanjiang, China; ^3^Guangzhou Key Laboratory of Materials for Energy Conversion and Storage, Guangdong Provincial Engineering Technology Research Center for Materials for Energy Conversion and Storage, School of Chemistry, South China Normal University (SCNU), Guangzhou, China; ^4^Institute of Advanced Interdisciplinary Studies, Chongqing University, Chongqing, China; ^5^Organic Semiconductor Research Center, Chongqing Institute of Green and Intelligent Technology, Chinese Academy of Sciences, Chongqing, China

**Keywords:** bulk heterojunction, polymer backbone fluorination, charge generation and recombination, vertical phase segregation, organic solar cells

In the published article, there was an error regarding the affiliations for Yuying Chang. As well as having affiliation(s) 1, they should also have “**Heilongjiang Province Key Laboratory of New Carbon-Base Functional and Superhard Material, Mudanjiang, China**.”

The authors apologize for this error and state that this does not change the scientific conclusions of the article in any way. The original article has been updated.

