# Supplementary material for: Impact of Polymer Backbone Fluorination on the Charge Generation/Recombination Patterns and Vertical Phase Segregation in Bulk Heterojunction Organic Solar Cells
Source: Front Chem. 2020 Mar 5;8:144. doi: 10.3389/fchem.2020.00144 (PMC7066253; doi:10.3389/fchem.2020.00144)
Supplement: Supplementary file 1 [file Data_Sheet_1.docx]

Supporting Information

Impact of Polymer Backbone Fluorination on the Charge Generation/Recombination Patterns and Vertical Phase Segregation in Bulk Heterojunction Organic Solar Cells

Yanqiu Shao*, Yuying Chang, Suju Zhang, Mingyue Bi, Shengjian Liu, Daliang Zhag, Shirong Lu, Zhipeng Kan*

**Content**

[**1.** **Device Preparation** 22](#_Toc32480670)

[**2.** **Additional PV Performance Data** 23](#_Toc32480671)

[**3.** **The polymer dispersion index and molecular weight** 24](#_Toc32480672)

[**4.** **The energy level diagram** 24](#_Toc32480673)

[**5.** **UV-vis Spectroscopy** 24](#_Toc32480674)

[**6.** **Charge Carrier Mobility** 25](#_Toc32480675)

[**7.** **Transient Characteristics** 27](#_Toc32480676)

[**8.** **EELS Analyses** 29](#_Toc32480677)

1. **Device Preparation**

The solar cells were prepared on glass substrates with tin-doped indium oxide (ITO, 15 Ω sq^−1^) patterned on the surface (device area: 0.1 cm^2^). Substrates were first scrubbed with dilute Extran 300 detergent solution to remove organic residues, and were then immersed in an ultrasonic bath of dilute Extran 300 for 30 min. The samples were rinsed under flowing deionized water for 5 min, and were then sonicated (Branson 5510) for 20 min each in successive baths of acetone and *iso*-propanol. Next, the samples were dried with pressurized nitrogen before being exposed to a UV−ozone plasma for 20 min. All solutions were prepared in a glovebox (N_2_) using the PBDT[2X]T polymers (with X=H or F) synthesized in-house by a previously reported method and PC_71_BM was purchased from SOLENNE. Optimized devices were fabricated by dissolving the polymers and PC_71_BM in chlorobenzene and adding 5% (v/v) of the processing additive 1-chloronaphthalene (CN). The solutions were prepared with a Polymer:PC_71_BM ratio of 1:1.5 (by weight), with a blend concentration of 20 mg mL^-1^ and were stirred for 4 h at 100 °C before being cast on the substrates. The active layers were spin-coated from the solutions at 90 °C at an optimized speed of 1000 rpm for 45 s, using a programmable spin coater from Specialty Coating Systems (Model G3P-8), resulting in films of 80 to 90 nm in thickness. The samples were then dried at room temperature for 1 h. Next, the samples were placed in a thermal evaporator for evaporation of a 5-nm thick calcium layer evaporated at 0.4 Å s^−1^ and a 100-nm thick aluminium electrode evaporated at 5 Å s^−1^; pressure of less than 2x10^-6^ Torr.

1. **Additional PV Performance Data**

**Table S1**. PV performance of the 2HT- and 2FT-based BHJ solar cells in normal device configuration.*^a,b,c^*

|  | *V_OC_* (V) | *J_SC_* (mA/cm^2^) | FF (%) | PCE (%) |
| --- | --- | --- | --- | --- |
| 2HT | 0.77±0.01 | 5.21±0.01 | 60.1±1.9 | 2.4±0.1 |
| 2FT | 0.89±0.01 | 9.32±0.16 | 73.3±1.0 | 6.1±0.1 |

*^a^*Optimized devices with 2HT-PC_71_BM and 2FT-PC_71_BM as active layers; solutions were preheated to *ca*. 90 ^o^C. *^b^*Average values across 10 devices (device area: 0.1 cm^2^); *^c^*Standard deviations calculated with the following equation (E1):

|  | $\sigma=\sqrt{\frac{1}{N}\sum_{i=1}^{N} \left( x_{i}-\mu\right)^{2}}$ | (E1) |
| --- | --- | --- |

where σ is the standard deviation, μ is the arithmetic average value of the devices’ PCE, N is the total number of devices used in the determination of the standard deviations.

**Table S2.** PV performance of the 2HT- and 2FT-based BHJ solar cells in inverted device configuration.*^a^*

|  | *V_OC_* (V) | *J_SC_* (mA/cm^2^) | FF (%) | PCE (%) |
| --- | --- | --- | --- | --- |
| 2HT | 0.60±0.01 | 5.18±0.01 | 50.8±1.9 | 1.6±0.1 |
| 2FT | 0.73±0.01 | 9.28±0.01 | 65.5±1.0 | 4.4±0.1 |

*^a^*Average values across 10 devices (device area: 0.1 cm^2^)


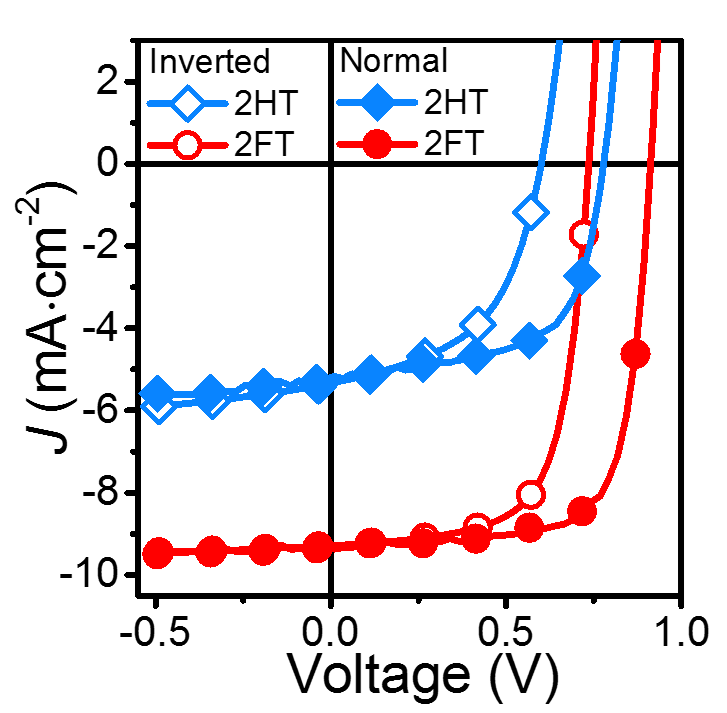


**Figure S1.** *J-V* characteristics of 2HT- and 2FT-based BHJ solar cells that gave the best performance in inverted device structures and normal device structures.

| Polymer donor | *M_n_*  (kDa) | *M_w_*  (kDa) | PDI |
| --- | --- | --- | --- |
| PBDT[2H]T | 15.5 | 33.2 | 2.1 |
| PBDT[2F]T | 12.6 | 25.8 | 2.0 |

1. **The polymer dispersion index and molecular weight**

**Table S3.** The polymer dispersion index and molecular weight of the PBDT[2X]T derivatives.

1. **The energy level diagram**

**Figure S2.** Energy level diagram of **PBDT[2H]T**, **PBDT[2F]T**, and **PC71BM**.

1. **UV-vis Spectroscopy**

**
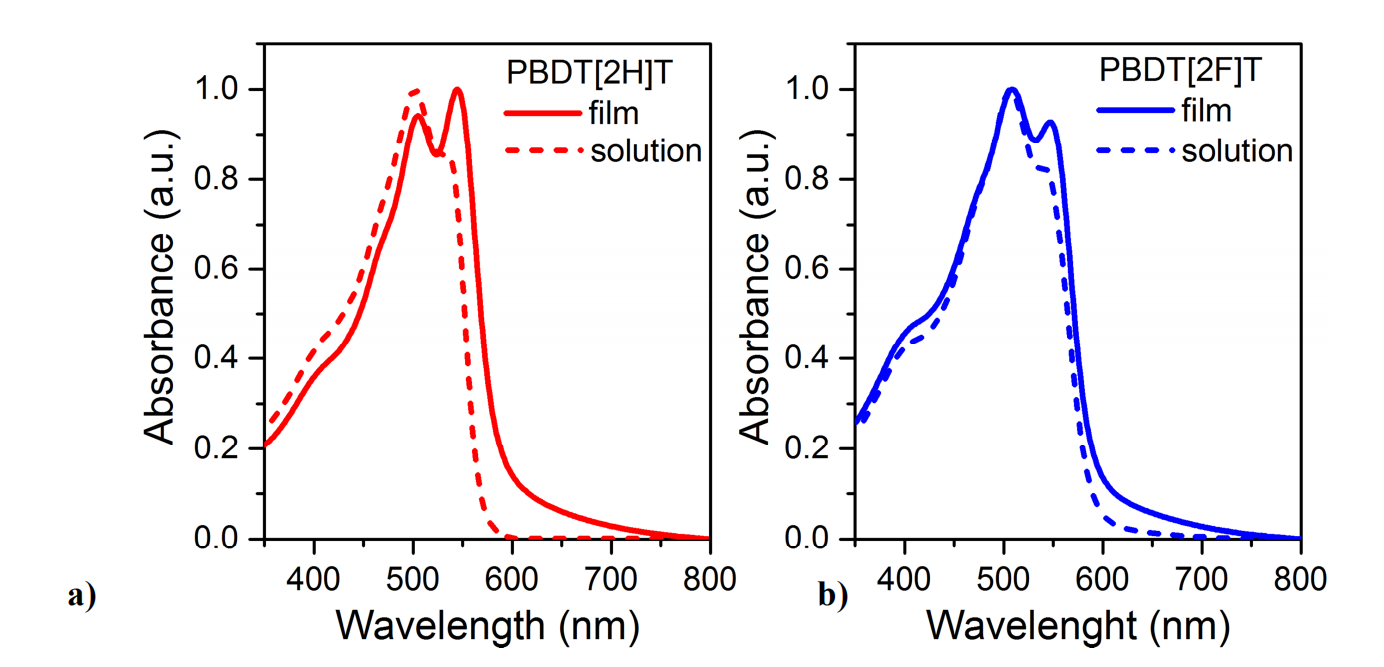
**

**Figure S3.** Normalized UV-vis spectra of (a) **PBDT[2H]T** and (b) **PBDT[2F]T** in chlorobenzene solution and for thin films cast from chlorobenzene solution onto glass substrates.

1. **Charge Carrier Mobility**

The carrier mobilities of the PBDT[2X]T: PC_71_BM were determined by fitting the dark current to the space-charge-limited current (SCLC) model using the following diode configurations: glass/ITO/MoO_3_/Active layer/MoO_3_/Ag and glass/ITO/Al/Active layer/Al for hole-only devices and electron-only devices, respectively. ITO substrates and solutions were prepared as described above. Film thicknesses were varied by using different spin-casting rates. In the hole-only device, molybdenum oxide (7 nm) was used as an electron-blocking layer and silver cathodes (100 nm) were thermally evaporated (~10^-6^ Torr) through a shadow mask defining an active area of 0.1 cm^2^. In the electron-only device, aluminum was used as both bottom and top electrodes. In both cases, the built-in voltage is 0 V because of the symmetrical transport layers.

**
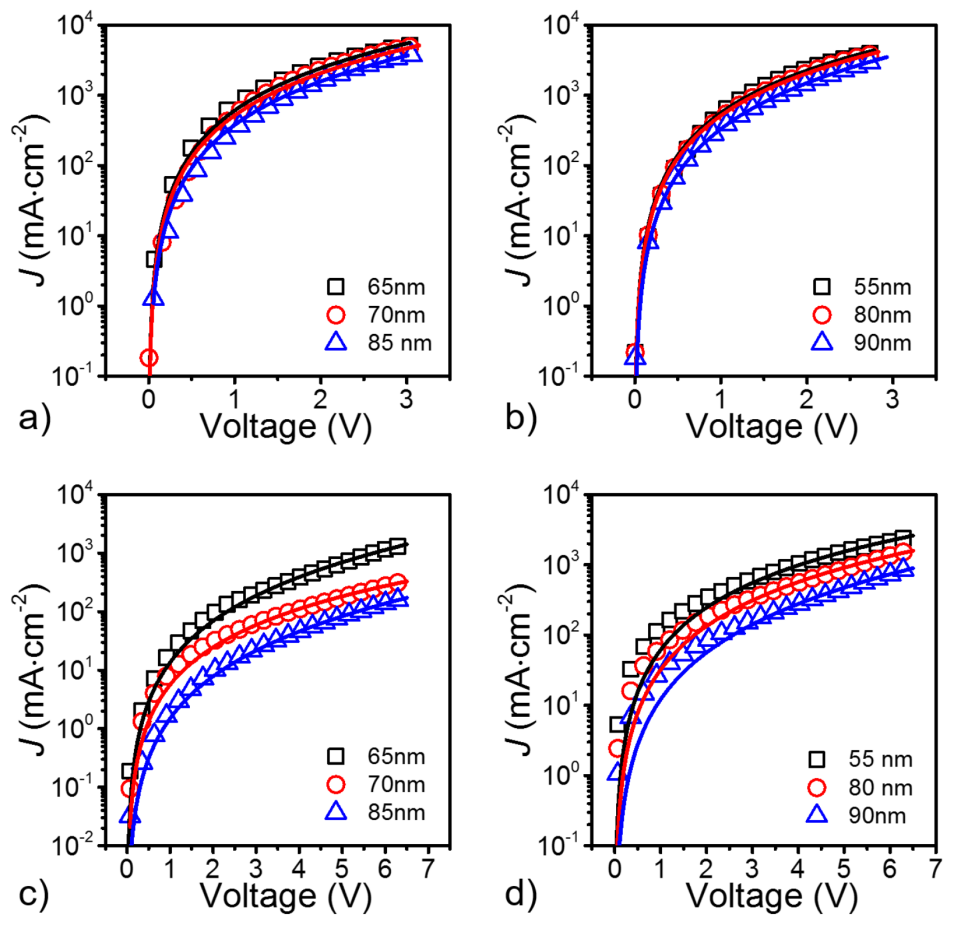
**

**Figure S4.** Dark *J - V* curves of PBDT[2X]T:PC_71_BM BHJ thin films of various thicknesses: electron-only diodes based on a) 2HT-based and b) 2FT-based diodes; hole-only diodes based on c) 2HT-based and d) 2FT-based diodes.

The parameters and their units in equation 1 are defined as in following table:

| Definition | Variable | Units |
| --- | --- | --- |
| zero-field mobility | *μ*_0_ | cm^2^ V^-1^ s^-1^ |
| film thickness | *L* | cm |
| dark current density | *J* | mA cm^-2^ |
| voltage | *V* | V |
| built in voltage | *V_bi_* | V |
| vacuum permittivity | *ε*_0_ (88.54 × 10^-12^) | mA s V^-1^ cm^-1^ |
| dielectric constant | *ε*_r_ (3) |  |
| field activation factor | *β* | cm^1/2^ V^-1/2^ |

**Table S4.** Hole mobility of the 2HT- and 2FT-based diodes as a function of the film thickness.

| Polymer donor | Film thickness (nm) | Zero-field mobility  (cm^2^ V^-1^ s^-1^ × 10^-4^) | Field activation factor  (× 10^-4^) | Adj. R-square |
| --- | --- | --- | --- | --- |
| PBDT[2H]T | 85 | 4.9 ± 0.2 | 5.2 | 0.99 |
|  | 70 | 5.3 ± 0.1 | 6.2 | 0.99 |
|  | 65 | 3.2 ± 0.4 | 10.5 | 0.99 |
| PBDT[2F]T | 90 | 2.1 ± 0.1 | 12 | 0.99 |
|  | 80 | 4.9 ±0.3 | 3.3 | 0.99 |
|  | 55 | 3.4 ± 0.2 | 0 | 0.99 |

**Table S5.** Electron mobility of the 2HT- and 2FT-based diodes as a function of the film thickness.

| Polymer donor | Film thickness (nm) | Zero-field mobility  (cm^2^ V^-1^ s^-1^ × 10^-4^) | Field activation factor  (× 10^-4^) | Adj. R-square |
| --- | --- | --- | --- | --- |
| PBDT[2H]T | 85 | 7.0 ± 0.2 | 3.3 | 0.99 |
|  | 70 | 5.4 ± 0.1 | 0 | 0.99 |
|  | 65 | 5.6 ± 0.4 | 0 | 0.99 |
| PBDT[2F]T | 90 | 7.5 ± 0.1 | 6.5 | 0.99 |
|  | 80 | 8.7 ±0.2 | 0.2 | 0.99 |
|  | 55 | 4.2 ± 0.2 | 0 | 0.99 |

1. **Transient Characteristics**

All the light intensity characteristics were performed using the all-in-one measurement system PAIOS 3.2 (Fluxim). PAIOS 3.2 employs many different device characterization techniques in steady-state and transient modes. A function generator controls the light source: a white LED (rise/fall time 100 ns). A second function generator controls the applied voltage. The current and the voltage of the solar cell are measured with a digitizer. The current is measured via the voltage drop over a 20 Ω resistor or a transimpedance amplifier, depending on the current amplitude.

The light source is a white LED with maximum power of 200 mW cm^-2^. Due to the spectral mismatch, this power does not represent 2 suns condition (AM 1.5). The 1 sun condition is determined by comparing the J_SC_ and V_OC_ measured with both Solar Simulator and PAIOS.

During the light intensity characteristics, in the case of J-V dependence on incident light intensity, the measured power was approximately from 0.17 up to 1.2 suns. When performing the transient photocurrent measurements, the pulse light was from the white LED source with a width of 200 μs with incident light intensity approximately from 0.02 up to 1 suns as shown in **Figure S5** and **Figure S6**. In the measurement of transient photovoltage, both the perturbation light pulse and background illumination light are again from the white LED. Data on TPC and TPV were collected by averaging 150 measurements under each incident light intensity. All the measurements were done on the best performing device of 2HT- or 2FT-based BHJ solar cells.


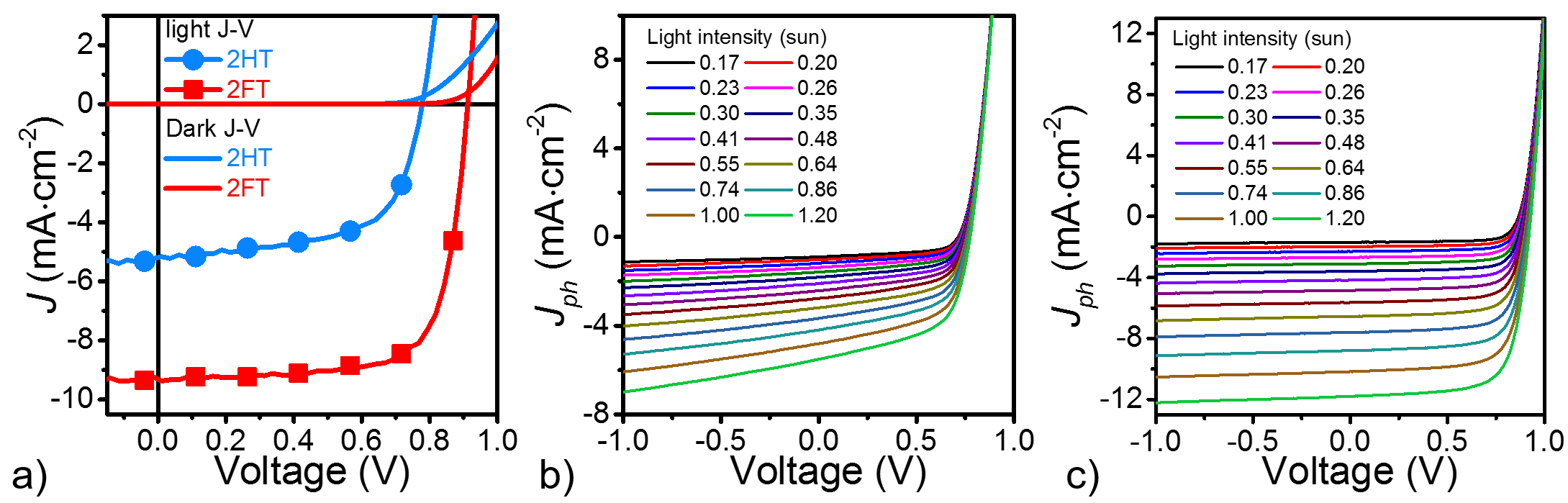


**Figure S5.** *J - V* characteristics of a) *J - V* under dark and with illumination, b) *J_ph_* of 2HT- based BHJ solar cells under different incident light intensity versus voltage, and c) *J_ph_* of 2FT- based BHJ solar cells under different incident light intensity versus voltage.


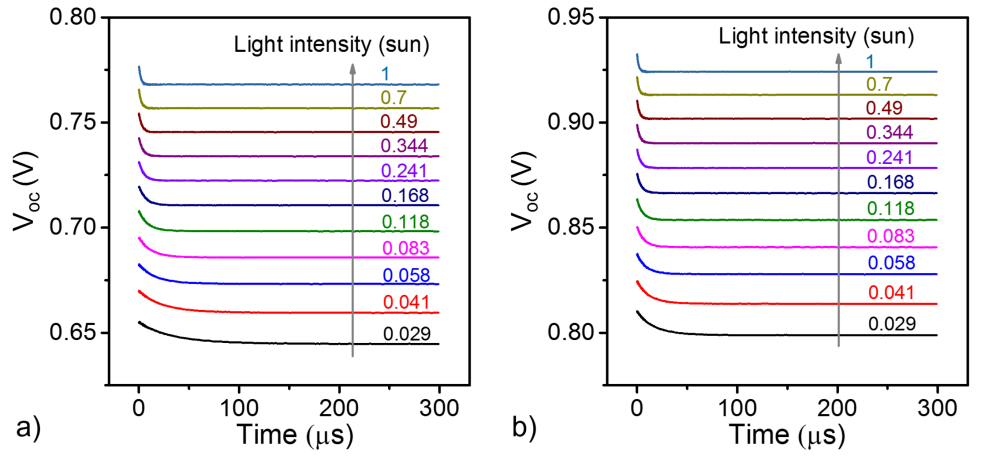


**Figure S6.** Transient photovoltage in response to a 200 µs white light (LED) pulse for (a) 2HT- and (b) 2FT-based BHJ solar cells, and the light intensity is given in sun equivalent.


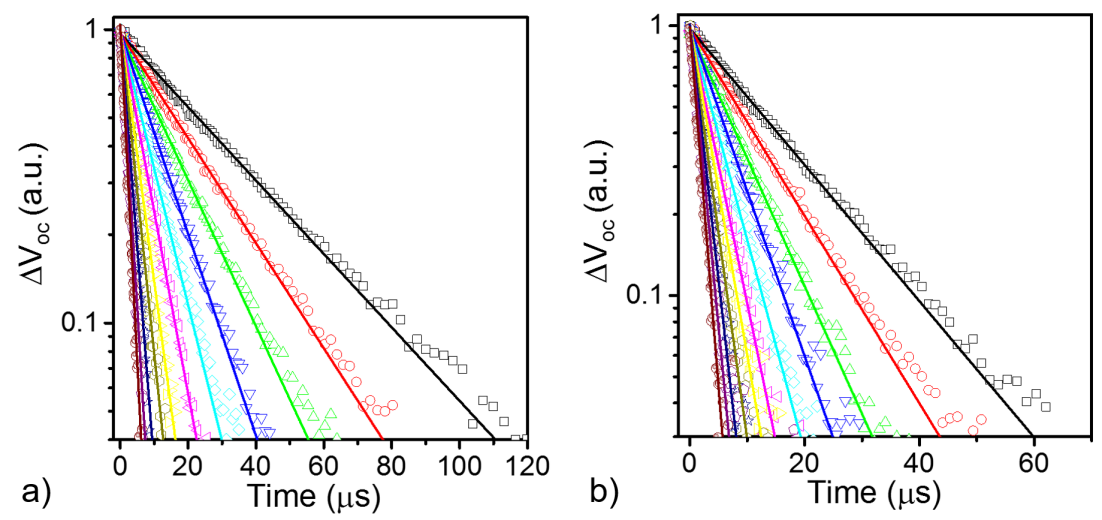


**Figure S7.** Normalized transient photovoltage in response to a 200 µs white light (LED) pulse for (a) 2HT- and (b) 2FT-based BHJ solar cells, and the solid lines are the mono-exponential fit to the experimental data. The corresponding incident light intensities are the same as in Figure S5.


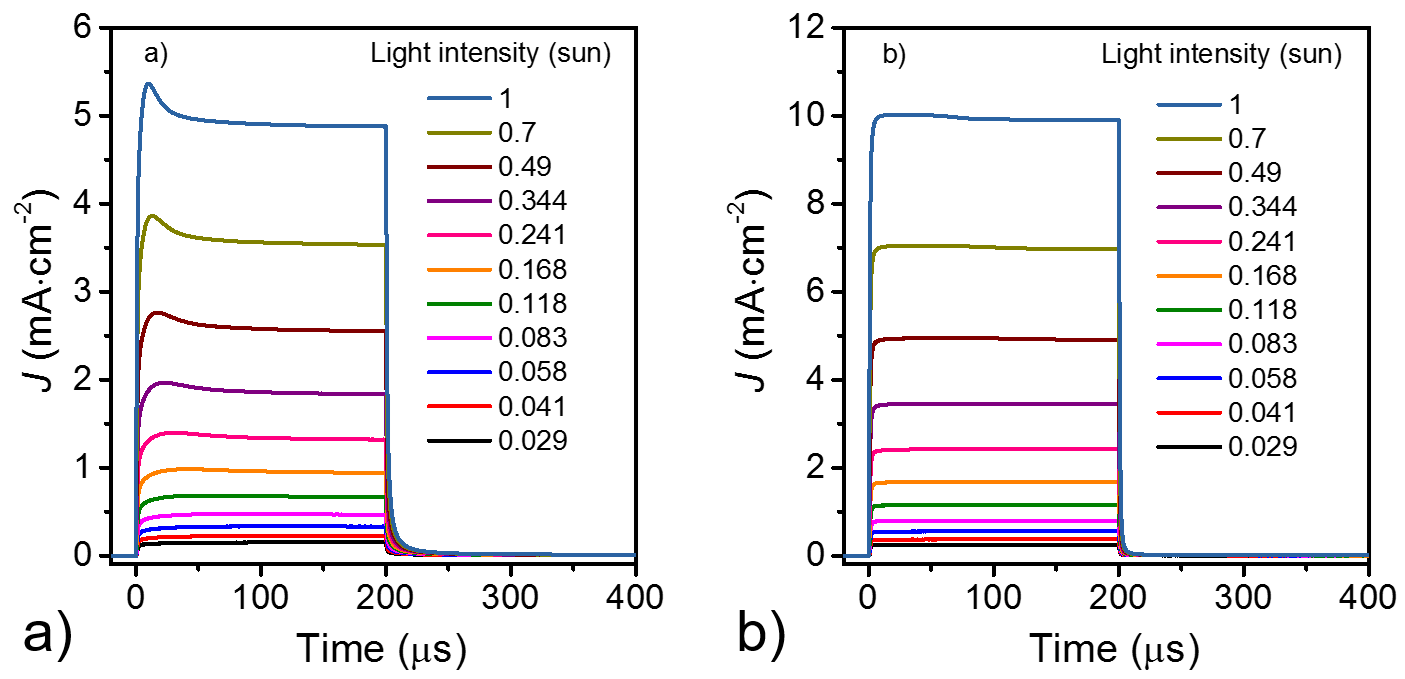


**Figure S8.** Transient photocurrent in response to a 200 µs white light (LED) pulse for (a) 2HT- and (b) 2FT-based BHJ solar cells, and the light intensity is given in sun equivalent.

1. **EELS Analyses**

TEM samples were prepared using an FEI Helios NanoLab 400S FIB/SEM dual-beam system equipped with a Ga+ ion source. Pt layers were deposited on the surface region of interested by Electron and Ion beam for protection. The sample was thinned down to a relative thickness of 80 nm using progressively decreasing ion beam energies in the FIB down to 2 keV.

The spectral mapping measurements were performed at 200 kV in STEM mode on an FEI Titan 80-300 Cube TEM equipped with probe aberration corrector and Gatan Quantum high resolution electron energy loss spectrometer. The STEM signals are collected by the Gatan Annular Dark-Field (ADF) detector. The S L2,3 edge is extracted from the EELS spectrum by MLS fitting method.
